# Ultrasonography indicators for predicting difficult intubation: a systematic review and meta-analysis

**DOI:** 10.1186/s12873-021-00472-w

**Published:** 2021-07-03

**Authors:** Mehran Sotoodehnia, Hosein Rafiemanesh, Hadi Mirfazaelian, Arash Safaie, Alireza Baratloo

**Affiliations:** 1 Prehospital and Hospital Emergency Research Center, Tehran University of Medical Sciences, Tehran, Iran; 2 Department of Emergency Medicine, Sina Hospital, Tehran University of Medical Sciences, Tehran, Iran; 3 Department of Epidemiology, School of Public Health and Safety, Shahid Beheshti University of Medical Sciences, Tehran, Iran; 4 Department of Emergency Medicine, Imam Khomeini Hospital Complex, Tehran University of Medical Sciences, Tehran, Iran

**Keywords:** Airway management, Difficult airway, Intratracheal intubation, Prediction, Ultrasonography

## Abstract

**Background:**

Ultrasonography (US) is recently used frequently as a tool for airway assessment prior to intubation (endotracheal tube (ETT) placement), and several indicators have been proposed in studies with different reported performances in this regard. This systematic review and meta-analysis reviewed the performance of US in difficult airway assessment.

**Methods:**

This systematic review and meta-analysis was conducted according to the guideline of Preferred Reporting Items for Systematic Reviews and Meta-Analyses (PRISMA) and the Cochrane book. All the studies that had carried out difficult airway assessments using US, had compared the indicators in difficult and easy groups, and had published the results in English by the time we conducted our search in April 28, 2020, were included.

**Results:**

In the initial search, 17,156 articles were retrieved. After deleting the duplicate articles retrieved from multiple databases, 7578 articles remained for screening based on the abstracts and titles. Finally, the full text of 371 articles were assessed and the data from 26 articles were extracted, which had examined a total of 45 US indicators for predicting difficult intubation. The most common US index was the “thickness of anterior neck soft tissue at the vocal cords level”. Also, “skin to epiglottis” and “anterior neck soft tissue at the hyoid bone level” were among the most common indicators examined in this area.

**Conclusion:**

This systematic review showed that US can be used for predicting difficult airway. Of note, “skin thickness at the epiglottis and hyoid levels”, “the hyomental distance”, and “the hyomental distance ratio” were correlated with difficult laryngoscopy in the meta-analysis. Many other indicators, including some ratios, have also been proposed for accurately predicting difficult intubation, although there have been no external validation studies on them.

## Background

Preparation is a key step in rapid sequence intubation (RSI) in emergency departments (EDs) and the assessment of difficult airways is an integral part of this procedure. Although physical examination and clinical criteria are used frequently for this purpose, there is still a 1.5% chance of difficult intubation (endotracheal tube (ETT) placement) with an increased rate in some populations, such as obese patients [1, 2]. This rare but ominous catastrophe is partially due to test flaws and variable inter-observer agreement [3, 4]. Furthermore, some rules might be difficult to apply in some settings with uncooperative patients, like those in EDs and critical care units [5]. Bedside ultrasonography (US) has recently been used in this regard. This safe, portable, and widely-available tool has been proposed for the assessment of airways [6]. Several indicators have been suggested in studies with different reported performances. This systematic review and meta-analysis reviewed the performance of US in difficult airway assessment.

## Methods

This study was conducted to systematically review studies that had assessed and compared US indicators in difficult and easy intubation group patients. The methods adopted for this systematic review and meta-analysis were consistent with the guideline of Preferred Reporting Items for Systematic Reviews and Meta-Analyses (PRISMA) and the Cochrane book.

### Search strategy

A comprehensive search was performed in international bibliometric databases including PubMed, ISI’s Web of Science, SCOPUS, and EMbase. The search terms were categorized and combined in two groups: Ultrasonography and airway evaluation. In the ultrasonography group, we used all possible keywords, such as sonograph, ultrasonic, Cormack lehane, hyposmia, and hypoxia. In the airway evaluation group, we used any possible keywords such as airway evaluation, airway management, airway investigation, difficult laryngoscopy, difficult airway, difficult intubation, endotracheal tube, endotracheal intubation, tracheal intubation, orotracheal intubation. The keywords were combined with the Boolean operator of “OR” in each group and with “AND” between the groups. No limitations were imposed in this study for publication time, and any article published by the time of the search in April 28, 2020, was included. The search strategy used in PubMed is presented in Appendix I. We completed our search by reviewing the references of the retrieved studies and contacting experts in this field in order to access further studies.

### Selection of studies and data extraction

The inclusion criteria for the studies were: 1) Having performed difficult airway assessment based on ultrasonography indicators, 2) Having compared the indicators in difficult and easy groups, and 3) Being published in English. The studies were excluded if they had used data from another included study or if their full text could not be accessed.

The identified documents were screened in two stages: 1) Screening the titles and abstracts to exclude the irrelevant studies and 2) Assessing the full texts for eligibility and inclusion criteria. Both stages were carried out independently by two reviewers, and discrepancies between the reviewers were resolved by a third reviewer. The full text was then reviewed to confirm that the eligibility criteria were met and for the extraction of the required information. Two researchers independently extracted the data of the included studies using a data extraction Excel-based sheet. The extracted data of each study were checked by two reviewers and discussed in the case of disagreements. The data extraction sheet included basic information (first author’s name, year of publication and country, design, participants, sampling method, and the demographic characteristics of the participants, such as age and BMI), difficult intubation, and sonographic indicators. All data on the US indicators, presented in two easy and difficult groups, were extracted by two of the researchers. The data included the range, mean, and standard deviation (SD) of sonographic indicators in the two groups. Also, the accuracy of US indicators for predicting or discriminating difficult intubation, such as the area under the ROC curve, best cut-off point, odds ratio (OR) and predictive values and likelihood ratios were extracted if they were reported. The quality of the studies was assessed using the Quality Assessment of Diagnostic Accuracy Studies, version 2 (QUADAS-2).

### Statistical analysis

All the eligible studies were included in the synthesis after their systematic review. We re-analyzed the raw data presented in one study (Wojtczak, J.A.; 2011) to obtain the mean of the US indicators in two easy and difficult groups. The mean difference of the US indicators in the two easy and difficult intubation groups were combined. The meta-analysis was conducted based on the random-effects model. The forest plot and pooled mean difference were presented for all the US indicators with at least two study. The heterogeneity of the preliminary studies was evaluated using the I-squared, Tau squared statistics, and Cochran’s *Q* test. The meta-analysis was performed in STATA statistical software, version 16 (StataCorp, College Station, TX).

## Results

### Screening and article selection

**Fig. 1:**
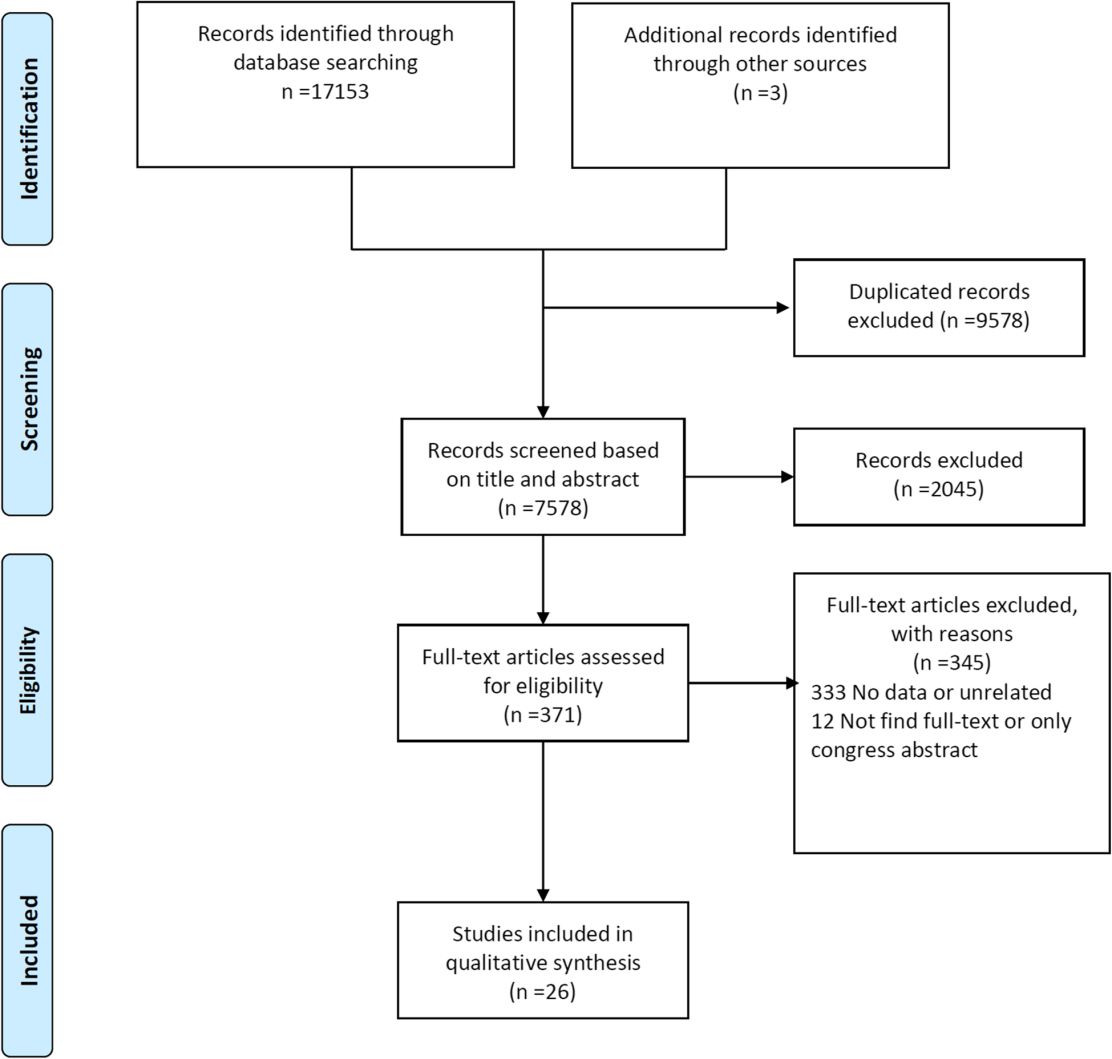
Flowchart of systematic review of ultrasonography indicators for predict of difficult intubation

The initial search yielded 17,156 articles. After deleting the duplicates, 7578 articles entered for screening based on their abstracts and titles. Finally, the full text of 371 articles was assessed and the data of 26 articles were extracted (Fig. 1). These articles had assessed at least one US index for patients with difficult intubation, laryngoscopy, or for different Cormack-Lehane grade groups.

### Characteristics of the included studies

The first article was published in 2003 and the majority of the articles had been published in recent years, with 16 (61.5%) published over the last 3 years (2017–2020). The 26 included studies were conducted in 11 different countries, with India (8 articles), China (6 articles), and the United States (4 articles) having the largest number of articles. The sample size of the studies ranged from 12 to 2254. The study by Yao, W. (2017) had the largest sample size and was conducted in China. This study was conducted on candidates of elective general surgery and anesthesia and assessed US indicators in groups of patients with and without difficult laryngoscopy and/or intubation. In the 26 studies we reviewed, the prevalence of difficult laryngoscopy and/or intubation was between 6.3 and 50%, and in the high-prevalence studies, the patients had been purposefully selected to compare US indicators and their high prevalence was therefore not generalizable (Table 1).

**Table 1:** Characteristics of included study in systematic review of ultrasonography indicators for predict of difficult intubation

First Author; Year	Country	Study design	Sample size	Participants	Demographic characteristics			Difficult intubation (%)
					Age	Sex (%)	BMI	
**Daggupati, H.; 2020** [7]	India	Prospective observational study	310	Patients posted for elective surgery planned under general anaesthesia with tracheal intubation using Macintosh laryngoscope	Mean (SD) = 33.0 (13.0)	M: 185 (59.7), F: 125 (40.3)	Mean (SD): 25.5 (2.1)	78 (25.0)
**Martínez-García, A.;2020** [8]	Spain	Prospective observational study	50	Adult patients, and ASA grade one to four, scheduled for surgery requiring general anesthesia with orotracheal intubation after classical laryngoscopy	Mean (SD) in Diff: 64.0 (11.0) vs Easy 52.0 (14.0)	M: 24 (48.0), F: 26 (52.0)	Mean (SD): Diff: 30.1 (3.5) vs Easy 30.0 (7.0)	16 (32.0)
**Fulkerson, J.S.;2019** [9]	USA	Cross-sectional, study	144	Veterans ages 19–79 scheduled for elective surgical procedures	Range = 29–78; Mean (SD) = 60.0 [10]	M: 130 (90.3), F: 14 (9.7)	Range = 17–46; Mean = 30.0	15 (10.4)
**Koundal, V.;2019** [11]	India	Prospective observational study	200	Patients requiring general anaesthesia and tracheal intubation	Mean = 43.2	M: 105 (52.5), F: 95 (47.5)	Mean = 22.2	29 (14.3)
**Wang, L.;2019** [12]	China	UK	508	Patients undergoing elective surgery under general anesthesia with tracheal intubation	Mean = 52.3	UK	Mean (SD) in Diff: 24.7 (0.49) vs Easy 23.5 (0.22)	47 (9.3)
**Xu, L.;2019** [10]	China	UK	119	Parturients undergoing elective cesarean delivery	Mean in Diff: 33.2 vs Easy 32.2	UK	Mean (SD) in Diff: 30.8 (3.6) vs Easy 27.2 (2.9)	40 (33.6)
**Yadav, N.K.;2019** [13]	India	Prospective single arm observational trial	310	Surgical patients, scheduled for various surgical procedures under general anaesthesia	Range = 18–70	UK	UK	35 (11.3)
**Abraham, S.; 2018** [14]	India	Prospective study	137	Patients underwent ultrasound followed by surgery under general anesthesia	Mean (SD) = 29.1 (10.5)	UK	UK	10 (7.3)
**Chan, S.M.M.; 2018** [15]	China	prospective clinical study	113	age of 18 years or above, who were scheduled for elective surgery requiring general anesthesia with direct laryngoscopy and tracheal intubation	Range = 19–84; Mean (SD) = 56.1 (12.9)	M = 69 (61.1), F: 44 (38.9)	BMI range: 15.6–52.2 BMI mean (SD): 24.5 (4.9)	39 (34.5)
**Falcetta, S.; 2018** [16]	Serbia	Prospective, single blinded, observational study	301	undergoing elective surgery under general anaesthesia with tracheal intubation	Mean (SD) = 57.2 (17.2)	M: 156 (51.8), F: 145 (48.2)	BMI mean: 25.8 (5.3)	28 (9.3)
**Petrișor, C.; 2018** [17]	Romania	Prospective observational	25	Patients with morbid obesity (Body Mass Index > 40 kg m-2, BMI), who needed to be operated on under general anaesthesia with oro-tracheal intubation	Mean (SD) in Diff: 52.0 (12.0) vs Easy: 46.0 (14.0)	In Diff: M: 1 (4.0), F: 3 (12.0) In Easy: M: 8 (32.0), F: 13 (52.0)	BMI mean (SD): Diff: 44.0 (7.6) vs Easy: 45.6 (0.79)	4 (16.0)
**Rana, S.; 2018** [18]	India	prospective, observational study	120	Patients scheduled for elective surgery requiring general anesthesia and tracheal intubation	Mean (SD) in CL1: 43.9 (12.0), CL2: 42.1 (15.3), CL3: 46.6 (9.4), CL4: 45.2 (7.1)	In total: M = 53 (44.2), F = 67 (55.8) In CL1: M: 13 (10.8), F:20 (16.7) In CL2: M: 24 (20.0), F:35 (29.2) In CL3: M: 10 (8.3), F:7 (5.8) In CL4: M:6 (5.0), F: 5 (4.2)	BMI mean (SD): CL1: 22.5 (12.6), CL2: 21.9 (23.2), CL3: 21.6 (20.4), CL4: 22.6 (11.7)	28 (12.5)
**Yilmaz, C.; 2018** [19]	Turkey	prospective, observational single-center study	74	aged > 18 years and morbidly obese (BMI > 35 kg/m2) who were scheduled for laparoscopic weight loss surgery under general anesthesia with endotracheal intubation	Mean (SD) in Diff: 43.3 (5.9) vs Easy 35.7 (8.7)	In Diff: M: 2 (2.7), F: 5 (6.8) In Easy: M: 8 (10.8), F: 59 (79.7)	BMI mean (SD): Diff: 45.2 (2.7) vs Easy: 47.6 (40.8)	7 (9.5)
**Parameswari, A.; 2017** [20]	India	prospective, double-blinded study	130	Patients scheduled for elective surgery requiring general anesthesia and surgery	Range = 18–60, Mean = 37.38 (SD = 12.756)	M: 63 (48.5), F:67 (51.5)	BMI range: 16.5–31.3	12 (9.2%)
**Yao, W.; 2017** [21]	China	prospective observational study	2254	Patients scheduled for elective surgery requiring general anesthesia and surgery	Mean (Range): In Lar. Diff: 61.0 (28–82) vs Easy: 49.0 (18–83) In Int. Diff: 61.0 (30–80) vs Easy: 50.0 (18–83)	Lar. Diff: M: 102 (4.5), F: 40 (1.8) Lar. Easy: M: 957 (42.5), F: 1155 (51.2) Int. Diff: M: 34 (1.5), F: 17 (0.8) Int. Easy: M: 1025 (42.5), F: 1178 (52.3)	BMI mean (SD): In Lar. Diff: 23.3 (3.3) vs Easy: 22.8 (3.5) In Int. Diff: 23.7 (3.7) vs Easy: 22.8 (3.5)	Diff. lar. =142 (6.3) / Diff. Int. = 51 (2.3)
**Yao, W.; 2017** [22]	China	prospective	484	elective surgery patients who were administered tracheal intubations under general anesthesia, ASA physical status I to III, and who were 18 to 90 years old.	Mean (SD) in Diff: 53.0 (14.0) vs Easy 48.0 (14.0)	In Diff: M: 29 (6.0), F: 12 (2.5) In Easy: M: 190 (39.3), F: 253 (52.3)	BMI mean (SD): Diff: 23.8 (3.1) vs Easy: 23.3 (3.6)	41 (8.47%)
**Andruszkiewicz, P.; 2016** [23]	Poland	prospective observational study	199	Patients scheduled for elective surgery requiring general anesthesia and surgery	Mean (SD) in Diff: 52.9 (8.3) vs Easy 51.3 (16.3)	In Diff: M: 10 (5.0), F: 12 (6.0) In Easy: M: 93 (46.7), F: 84 (42.2)	BMI mean (SD): Diff: 27.3 (6.9) vs Easy: 26.7 (5.0)	22 (11.1)
**Pinto, J.; 2016** [24]	Portugal	prospectivedouble blind study	74	ASA class I - III scheduled surgical/ Pregnant & morbid obesit was excluded	Mean (SD) in Diff: 57.5 (11.1) vs Easy: 55.2 (18.1)	In Diff: M: 13 (17.6), F: 4 (5.4) In Easy: M: 26 (35.1), F: 31 (41.9)	BMI mean (SD): Diff: 28.9 (4.7) vs Easy: 27.5 (5.2)	17 (22.97)
**Reddy, P.B.; 2016** [25]	India	prospective, observational study	100	Patients scheduled for elective surgery requiring general anesthesia and surgery	Range = 18–70	M: 69 (69.0), F: 31 (31.0)	BMI range: 14.2–39.0	14 (14.0)
**Hui, C.M.; 2014** [26]	Canada	UK	100	Adult patients (> 17 years old) presenting for elective surgery and requiring routine tracheal intubation	Mean (SD) = 52.1 (15.5)	M: 55 (55.0), F: 45 (45.0)	BMI mean (SD): 28.4 (5.3)	11 (11.0)
**Wu, J.; 2014** [27]	China	prospective observational study	203	age 20–65 years scheduled to undergo general anesthesia ASA I II	Mean (SD) in Diff: 46.0 (15.0) vs Easy 47.0 [14]	In Diff: M: 14 (6.9), F: 14 (6.9) In Easy: M: 69 (34.0), F: 106 (52.2)	BMI mean (SD): Diff: 25.6 (2.8) vs Easy: 23.6 (3.4)	28 (13.79)
**Gupta, D.; 2013** [28]	India	UK	49	patients scheduled for elective surgery and requiring general anesthesia with direct laryngoscopy and endotracheal intubation	UK	UK	UK	12 (24.49)
**Adhikari, S.; 2011** [5]	USA	prospective observational study	51	Adult patients undergoing endotracheal intubation for an elective surgical procedure	Mean (SD) = 53.1 (13.2)	M: 19 (37.3), F: 32 (62.7)	UK	12 (23.5)
**Wojtczak, J.A.; 2011** [29]	USA	UK	12	Five obese and 7 morbidly obese adult patients with a history of either difficult or easy intubation	Mean in Diff: 36.8 vs Easy. Mean = 36.8	M: 7 (58.3), F: 5 (41.7)	BMI range: 30.1–52.3, 7patient > 40	6 (50.0)
**Komatsu, R.; 2007** [30]	USA	UK	64	morbidly obese patients (BMI > 35) scheduled for elective surgery under general anaesthesia with tracheal intubation	Mean (SD) in Diff: 47.0 (9.0) vs Easy: 42.0 (11.0)	In Diff: M: 3 (4.7), F: 17 (26.6) In Easy: M: 9 (14.1), F: 35 (54.7)	BMI mean (SD): Diff: 56.0 (12.0) vs Easy: 57(SD = 15)	20 (31.3)
**Ezri, T.; 2003** [31]	Israel	UK	50	50 morbidly obese patients (BMI > 35 kg·m − 2) scheduled for laparoscopic weight reduction surgery (LapBand) undergeneral anaesthesia with endotracheal intubation	Mean (SD) in Diff: 33.6 (6.0) vs Easy 38.8 (8.4)	In Diff: M: 7 (14.0), F: 2 (4.0) In Easy: M: 14 (28.0), F: 27 (54.0)	BMI mean (SD): Diff: 44.0 (4.8) vs Easy: 43.0 (4.0)	9 (18.0)

*BMI* Body mass index, *CL* Cormack-Lehane grade, *Diff* Difficult intubation group, *SD* Standard deviation, *M* male, *F* Female, *UK* Un-Known

We used QUADA-2 for assessing the quality of the studies (Table 2). Patient selection bias was the most common bias encountered in the reviewed studies. Four studies were judged to have a high risk for patient selection bias due to their convenience sampling method [9], unclear recruitment method and small sample size [29], unknown recruitment method, too many exclusion criteria, and undisclosed excluded cases [31], or unknown recruitment method and including only parturient women [10]. Sixteen studies were deemed to have an unclear risk of patient selection bias due to their unclear recruitment strategy [8, 11–18, 20–22, 24, 27, 28, 30]. All of the studies were judged to have a low risk of index test bias. Seven studies were deemed to have an unclear risk of reference test bias because data about intubator blinding was not disclosed by them [5, 14, 17, 20, 27, 29, 31]. One study was judged to have a high risk of flow and timing bias due to performing airway ultrasonography 5–10 days after the intubation [23]. All of the studies were deemed to have low applicability concerns, but one study was at risk for high applicability concerns due to performing laryngoscopy in sedated non-paralyzed patients [10].

**Table 2:** Baseline characteristics of selected studies

Study	Risk of bias				Applicability concerns		
	Patient selection	Index test	Reference standard	Flow and timing	Patient selection	Index test	Reference standard
**Daggupati, H.; 2020** [7]	Low	Low	Low	Low	Low	Low	Low
**Martínez-García, A.;2020** [8]	Unclear	Low	Low	Low	Low	Low	Low
**Fulkerson, J.S.;2019** [9]	High	Low	Low	Low	Low	Low	Low
**Koundal, V.;2019** [11]	Unclear	Low	Low	Low	Low	Low	Low
**Wang, L.;2019** [12]	Unclear	Low	Low	Low	Low	Low	Low
**Xu, L.;2019** [10]	High	Low	Low	Low	Low	Low	High
**Yadav, N.K.;2019** [13]	Unclear	Low	Low	Low	Low	Low	Low
**Abraham, S.; 2018** [14]	Unclear	Low	Unclear	Low	Low	Low	Low
**Chan, S.M.M.; 2018** [15]	Unclear	Low	Low	Low	Low	Low	Low
**Falcetta, S.; 2018** [16]	Unclear	Low	Low	Low	Low	Low	Low
**Petrișor, C.; 2018** [17]	Unclear	Low	Unclear	Low	Low	Low	Unclear
**Rana, S.; 2018** [18]	Unclear	Low	Low	Low	Low	Low	Low
**Yilmaz, C.; 2018** [19]	Low	Low	Low	Low	Low	Low	Low
**Parameswari, A.; 2017** [20]	Unclear	Low	Unclear	Low	Low	Low	Low
**Yao, W.; 2017** [21]	Unclear	Low	Low	Low	Low	Low	Low
**Yao, W.; 2017** [22]	Unclear	Low	Low	Low	Low	Low	Low
**Andruszkiewicz, P.; 2016** [23]	Low	Low	Low	High	Low	Low	Low
**Pinto, J.; 2016** [24]	Unclear	Unclear	Low	Low	Low	Low	Low
**Reddy, P.B.; 2016** [25]	Low	Low	Low	Low	Low	Low	Low
**Hui, C.M.; 2014** [26]	Low	Low	Low	Low	Low	Low	Low
**Wu, J.; 2014** [27]	Unclear	Low	Unclear	Low	Low	Low	Low
**Gupta, D.; 2013** [28]	Unclear	Low	Unclear	Low	Low	Low	Low
**Adhikari, S.; 2011** [5]	Low	Low	Low	Low	Low	Low	Low
**Wojtczak, J.A.; 2011** [29]	High	Low	Unclear	Low	Low	Low	Low
**Komatsu, R.; 2007** [30]	Unclear	Low	Low	Low	Low	Low	Low
**Ezri, T.; 2003** [31]	High	Low	Unclear	Low	Low	Low	Low

### Predictive ultrasonography indicators for difficult intubation

A total of 45 US indicators for predicting difficult intubation were examined in the 26 reviewed studies. The most common assessed ultrasound criterion was “thickness of the anterior neck soft tissue at the vocal cords”, which was studied in nine different studies between 2003 and 2020. Also, “skin to epiglottis” and “anterior neck soft tissue at the hyoid bone” were among the most common US indicators examined in eight different studies. The following are the results of each of the indicators.

#### Distance from the skin to the epiglottis

**Fig. 2:**
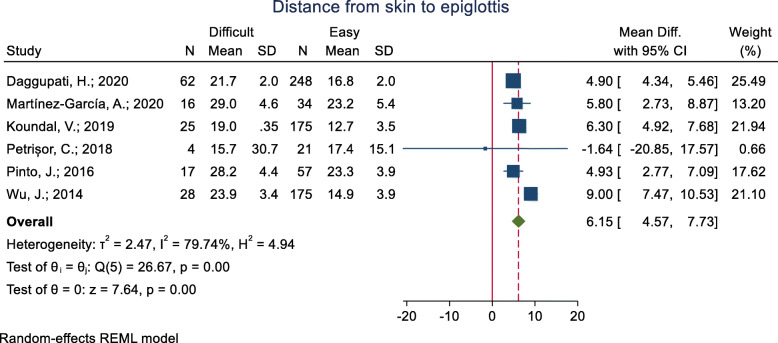
Forest-plot and pooled mean difference of distance from skin to epiglottis index in difficult and easy intubation group

This criterion has been examined in eight studies (Table 3). The mean of this index was assessed in six studies. In five of these studies, the mean of distance from the skin to the epiglottis was significantly higher in the difficult group (*p* < 0.05), and in the other study, it was higher in the easy group, although the difference was not significant. The pooled mean difference of distance from the skin to the epiglottis based on the meta-analysis results was 6.15 mm higher in the difficult group than the easy group, and the difference was statistically significant (*p* < 0.001) (Fig. 2). The AUC reported was 0.79 and 0.91 in four studies. In three of these studies, the optimal cut-off point calculated was 1.62 (sensitivity = 89.7 and specificity = 64.8), 2.54 (sensitivity = 82.0 and specificity = 91.0) and 3.0 (sensitivity = 56.3 and specificity = 88.2). In the other one, accuracy indicators were reported, but the cut-off point was not.

**Table 3:** The ultrasonography indicators and main result extracted for predict of difficult intubation

	Indicators list in study	Presented in	Main result (Mean difference, accuracy, and other results)
Distance from skin to epiglottis	Skin to epiglottis distance (cm)	Daggupati, H.; 2020	**Mean (95% CI):** In Diff = 2.17 (2.12–2.22) vs Easy = 1.68 (1.65–1.70); *P*-value< 0.001
	Neck soft tissue from skin to epiglottis (DSE) (cm)	Martínez-García, A.;2020	**Mean (SD):** In Diff = 2.90 (0.46) vs Easy = 2.32 (0.54); *P*-value = 0.001 **AUC** = 0.79 (95%CI: 0.62–0.89), P = 0.001**; In cut-off = UK:** Sensitivity =93.75%، Specificity =50.11%، PPV =46.88%، NPV =94.44% **In cut-off = 3:** Sensitivity = 56.3% (95% CI: 28.8–83.7), Specificity = 88.2% (95% CI: 75.9–100), PPV = 69.2% (95% CI: 40.3–98.2), NPV = 81.1% (95% CI: 67.1–95.1)
	Distance from skin to epiglottis (DSEM)	Koundal, V.;2019	**Mean (SD):** In CL1 = 1.42 (0.33), CL2 = 1.46 (0.36), CL3 = 1.89 (0.36), CL4 = 1.96 (0.21); *P*-value< 0.001 **AUC** = 0.819 (95%CI: 0.758–0.880), **In cut-off = 1.615:** Sensitivity = 89.7%, Specificity = 64.8%, PPV = 50.98%, NPV = 93.88%
	Pre-epiglottic Soft tissue thickness (mm)	Petrișor, C.; 2018	**Mean (SD):** In Diff = 15.75 (30.73) vs Easy = 17.39 (15.15); P-value = 0.6
	Distance from skin to epiglottis (DSE) (mm)	Pinto, J.; 2016	**Mean (SD):** In Diff = 28.25 (4.43) vs Easy = 23.32 (3.86); P-value = 0.000
	At thyrohyoid membrane level, the distance from skin to epiglottis midway (DSEM) (cm)	Wu, J.; 2014	**Mean (SD):** In Diff = 2.39 (0.34) vs Easy = 1.49 (0.39); P-value< 0.001 **AUC** = 0.90 (95% CI: 0.85–0.94); *P*-value< 0.001
	mDSE (median distance from skin to epiglottis), (cm)	Falcetta, S.; 2018	**AUC** = 0.906 (95% CI: 0.86–0.93), **In cut-off = 2.54:** Sensitivity = 82.0%, Specificity = 91.0%
	Skin to epiglottis	Parameswari, A.; 2017	**In cut-off = UK:** Sensitivity = 75.0%, Specificity = 63.6%, PPV = 17.5, NPV = 96.2
Thickness of anterior neck soft tissue at Vocal cords	Anterior neck thicknesses at the Vocal cords	Fulkerson, J.S.;2019	**Mean (SD):** In Diff = 0.73 (0.15) vs Easy = 0.70 (0.23); *P*-value = 0.631
	Distance from skin to glottis	Martínez-García, A.;2020	**Mean (SD):** In Diff = 1.05 (0.25) vs Easy = 1.07 (0.33); *P*-value = 0.749 **AUC** = 0.47 (95%CI: 0.31–0.64), *P* = 0.755**; In cut-off = UK:** Sensitivity =81.25%، Specificity =23.53%، PPV =33.33%، NPV =72.73%
	Thickness of anterior neck soft tissue at Vocal cords	Adhikari, S.; 2011	No-significant difference in Diff and Easy
	Neck soft tissue, from the skin to the anterior aspect of the trachea at the vocal cords anterior to the thyroid cartilage	Komatsu, R.; 2007	**Mean (SD):** In Diff = 20.4 (3.0) vs Easy = 22.3 (3.8); P-value = 0.049 **OR** = 0.16 (0.02–1.75); *P*-value = 0.134
	Distance from the skin to the anterior aspect of the trachea was measured at vocal cords (zone 1)	Yilmaz, C.; 2018	**Mean (SD):** In Diff = 1.21 (0.28) vs Easy = 1.32 (0.30); P-value = 0.260 **OR** = 0.204 (95% CI: 0.006–7.34; *P*-value = 0.385
	At anterior commissure level, the minimal distance from skin to anterior commissure (DSAC)	Wu, J.; 2014	**Mean (SD):** In Diff = 1.30 (0.31) vs Easy = 0.92 (0.20); P-value< 0.001 **AUC** = 0.85 (95% CI: 0.79–0.89); *P*-value< 0.001
	The distance from the skin to the anterior aspect of the trachea was measured at vocal cords (zone 1)	Ezri, T.; 2003	**Mean (SD):** In Diff 28.0 (2.7) vs Easy 17.5 (1.8); P-value< 0.001
	mVC (the median distance from the skin to the apex of the vocal cords),	Falcetta, S.; 2018	**AUC** = 0.54 (95% CI:0.48–0.60), Sensitivity =53, Specificity =66
	Anterior neck soft tissue thickness at the level of the vocal cords (ANS-VC)	Reddy, P.B.; 2016	**Mean (SD):** In CL1 = 0.25 (0.11), Range = 0.11–0.53/ CL2 = 0.25 (0.12), Range = 0.07–0.67/ CL3 = 0.35 (0.18), Range = 0.18–0.76; *P*-value = 0.014
Anterior neck soft tissue at the of the hyoid bone	Neck soft tissue from skin to hyoid (DSH) (cm)	Martínez-García, A.;2020	**Mean (SD):** In Diff = 1.35 (0.21) vs Easy = 1.30 (0.31); P-value = 0.580 **AUC** = 0.57 (95%CI: 0.40–0.73); *P* < 0.001**; In cut-off = UK:** Sensitivity =75.01%، Specificity =41.18%، PPV =37.5%، NPV =77.78%
	Anterior neck thicknesses at the hyoid Bone (HB) (cm)	Fulkerson, J.S.;2019	**Mean (SD):** In Diff = 0.93 (0.22) vs Easy = 0.97 (0.31); P-value = 0.681
	Thickness of anterior soft tissue neck at the level of hyoid bone (DSHB)	Koundal, V.;2019	**Mean (SD):** In CL1 = 0.84 (0.16), CL2 = 0.85 (0.17), CL3 = 0.98 (0.23), CL4 = 1.15 (0.18); P-value< 0.001 **AUC** = 0.680 (95%CI: 0.594–0.767), **In cut-off = 0.99:** Sensitivity = 48.0%, Specificity = 82.0%, PPV = 52.83%, NPV = 79.59%
	Distance from skin to hyoid bone (SHB) in neutral	Yadav, N.K.;2019	**Mean (SD):** In Diff = 0.74 (0.23) vs Easy = 0.56 (0.19); *P*-value = 0.001 **AUC** = 0.72 (95%CI: 0.61–0.82), **In cut-off = 0.66:** Sensitivity = 68.0%, Specificity = 69.0%
	Anterior neck soft tissue at the of the hyoid bone	Adhikari, S.; 2011	**Mean (CI 95%):** In Diff = 1.69 cm (1.19–2.19) vs Easy = 1.37 (1.27–1.46); *P*-value< 0.05
	At hyoid bone level, the minimal distance from the hyoid bone to skin surface (DSHB) (cm)	Wu, J.; 2014	**Mean (SD):** In Diff = 1.51 (0.27) vs Easy = 0.98 (0.26); P-value< 0.001 **AUC** = 0.92 (95%CI: 0.87–0.95); P < 0.001
	Skin to hyoid distance	Parameswari, A.; 2017	**In cut-off = UK:** Sensitivity = 58.3%, Specificity = 56.8%, PPV = 12.1, NPV = 93.1
	Anterior neck soft tissue thickness at the level of the hyoid (ANS-Hyoid)	Reddy, P.B.; 2016	**Mean (SD):** In CL1 = 0.36 (0.20), Range = 0.12–0.98/ CL2 = 0.35 (0.14), Range = 0.15–0.69/ CL3 = 0.38 (0.16), Range = 0.18–0.68; *P*-value = 0.857
Hyomental distance (HMD) with neck extended	HMD distance between the hyoid bone and the posterior border of the symphisis menti: maximum hyperextended	Petrișor, C.; 2018	**Mean (SD):** In Diff 4.9 (0.22) vs Easy 5.8 (0.42); P-value = 0.1
	Hyomental distance (HMD) with neck extended (cm)	Fulkerson, J.S.;2019	**Mean (SD):** In Diff = 5.10 (0.65) vs Easy = 5.28 (0.69); *P*-value = 0.341
	Mentohyoid distance	Daggupati, H.; 2020	**Mean (95% CI):** In Diff = 3.70 (3.5–3.9) vs Easy = 4.72 (4.63–4.90); *P*-value = 0.341
Hyomental distance ratio	Hyomental distance ratio (HMDR) (cm)	Koundal, V.;2019	**Mean (SD):** In CL1 = 1.12 (0.03), CL2 = 1.11 (0.03), CL3 = 1.09 (0.01), CL4 = 1.04 (0.02); P-value< 0.001 **AUC** = 0.762 (95%CI: 0.686–0.838), **In cut-off = 1.087:** Sensitivity = 65.0%, Specificity = 77.0%, PPV = 54.29%, NPV = 84.62%
	HMDR (the ratio between the HMD in the maximum hyperextended position to that in the neutral position)	Petrișor, C.; 2018	**Mean (SD):** In Diff 1.21 (0.0005) vs Easy 1.34 (0.1); *P*-value = 0.0002
	Hyomental distance ratio (HMDR) (cm)	Wojtczak, J.A.; 2011	**Mean (SD):** In Diff 1.02 (0.02) vs Easy 1.14 (0.02); P-value< 0.001
	Hyomental distance ratio (HMDR), cm	Rana, S.; 2018	**Mean (SD):** In CL1 = 1.11 (0.35), CL2 = 1.12 (0.29), CL3 = 1.07 (0.39), CL4 = 1.04 (0.01); *P*-value< 0.001 **AUC** = 0.871, **In cut-off = 1.085:** Sensitivity = 75.0%, Specificity = 85.3%, PPV = 65.6%, NPV = 90.1%
	Hyomental distance ratio (HMDR) (cm)	Andruszkiewicz, P.; 2016	**Mean (SD):** In Diff = 1.07 (0.08) vs Easy = 1.12 (0.07); P-value = 0.0022 **AUC** = 0.710, *P*-value = 0.0036; **In cut-off = UK:** Sensitivity = 42.9%, Specificity = 96.0%, PPV = 56.2%, NPV = 93.4%;
Pre-E/EVC	Pre-E/E-VC	Koundal, V.;2019	**Mean (SD):** In CL1 = 1.22 (0.44), CL2 = 0.56 (0.27), CL3 = 1.91 (0.25), CL4 = 2.25 (0.31); P-value< 0.001 **AUC** = 0.871 (95%CI: 0.820–0.923), **In cut-off = 1.875:** Sensitivity = 82.8%, Specificity = 83.8%, PPV = 67.61%, NPV = 92.25%
	Pre-E/EVC (depth of the pre-epiglottic space/ the distance from the epiglottis to the midpoint of the distance between the vocal cords)	Rana, S.; 2018	**Mean (SD):** In CL1 = 1.33 (0.335), CL2 = 1.62 (0.264), CL3 = 1.87 (0.243), CL4 = 2.22 (0.29); *P*-value< 0.001 AUC = 0.868, **In cut-off = 1.77:** Sensitivity = 82.0%, Specificity = 80.0%, PPV = 60.5%, NPV = 92.3%
	Pre-E/E-VC	Reddy, P.B.; 2016	**Mean (SD):** In CL1 = 1.09 (0.38), Range = 0.41–2.22/ CL2 = 1.28 (0.37), Range = 0.58–2.02/ CL3 = 1.29 (0.44), Range = 0.76–2.46; P-value = 0.044
	Ratio of Pre-Epiglottis space and Epiglottis-to-Vocal cords distances (Pre-E/E-VC)	Gupta, D.; 2013	**Mean (SD):** In CL1 = 0.89 (0.61), CL2 = 1.65 (0.81), CL3 = 2.54 (0.98), CL4 = No data yet, P-value = UK
Anterior neck soft tissue at thyroid isthmus	Thickness of anterior neck soft tissue at thyroid isthmus	Adhikari, S.; 2011	No-significant difference in Diff and Easy
	Distance from the skin to the anterior aspect of the trachea was measured thyroid isthmus	Yilmaz, C.; 2018	**Mean (SD):** In Diff = 1.55 (0.32) vs Easy = 1.78 (0.39); P-value = 0.130 **OR** = 0.144 (95% CI: 0.008–2.56); *P*-value = 0.187
	The distance from the skin to the anterior aspect of the trachea was measured at thyroid isthmus (zone 2)	Ezri, T.; 2003	**Mean (SD):** In Diff = 25.0 (1.3) vs Easy = 22.8 (5.0); P-value = 0.16
Anterior neck soft tissue at suprasternal notch	Thickness of anterior neck soft tissue at Suprasternal notch	Adhikari, S.; 2011	No-significant difference in Diff and Easy
	Distance from the skin to the anterior aspect of the trachea was measured at suprasternal notch (zone 3)	Yilmaz, C.; 2018	**Mean (SD):** In Diff = 2.26 (0.55) vs Easy = 2.32 (0.52); P-value = 0.875 **OR** = 0.924 (95% CI: 0.15–5.56); *P*-value = 0.931
	The distance from the skin to the anterior aspect of the trachea was measured at suprasternal notch (zone 3)	Ezri, T.; 2003	**Mean (SD):** In Diff = 33.0 (4.3) vs Easy = 27.4 (6.6); P-value = 0.013
Tongue volume	Tongue volume (cm3)	Wojtczak, J.A.; 2011	**Mean (SD):** In Diff = 137.67 (29.28) vs Easy = 168.33 (34.22); *P*-value = 0.126
	Volume of tongue, cm3	Parameswari, A.; 2017	**In cut-off = 100:** Sensitivity = 66.7%, Specificity = 62.7%, PPV = 15.4%, NPV = 94.6%
	Tongue volume	Andruszkiewicz, P.; 2016	**Mean (SD):** In Diff = 121.7 (27.1) vs Easy = 111.2 (22.1); P-value = 0.0415 AUC = 0.626, *P*-value = 0.0456; **In cut-off = UK:** Sensitivity = 9.1%, Specificity = 97.7%, PPV = 33.3%, NPV = 89.6%;
Floor of the mouth muscle volumes	Floor of the mouth muscle volumes (MVFM, muscle volume of the floor of the mouth (cm3))	Wojtczak, J.A.; 2011	**Mean (SD):** In Diff = 34.87 (11.95) vs Easy = 37.72 (13.17); P-value = 0.703
	Volume of floor of mouth	Parameswari, A.; 2017	**In cut-off = UK:** Sensitivity = 50.0%, Specificity = 55.9%, PPV = 10.3, NPV = 91.7
	floor of the mouth muscle volume (FMMV) (cm3)	Andruszkiewicz, P.; 2016	**Mean (SD):** In Diff = 20.10 (5.39) vs Easy = 19.32 (4.15); *P*-value = 0.4224 **AUC** = 0.559, *P*-value = 0.421; **In cut-off = UK:** Sensitivity = 31.7%, Specificity = 71.8%, PPV = 12.3%, NPV = 89.4%
Hyomental distance in the head positions	Hyomental distance in the head positions (HMDE) (mm)	Wojtczak, J.A.; 2011	**Mean (SD):** In Diff = 52.65 (5.89) vs Easy = 65.65 (4.17); *P*-value = 0.001
	hyomental distance in extension positions (HMDE) (cm)	Andruszkiewicz, P.; 2016	**Mean (SD):** In Diff = 4.28 (0.64) vs Easy = 4.82 (0.46); P-value = 0.0009 **AUC** = 0.758, *P*-value< 0.0001; **In cut-off = UK:** Sensitivity = 38.1%, Specificity = 97.7%, PPV = 66.7%, NPV = 93.0%
Hyomental distance in the neutral positions	Hyomental distance in the neutral positions (HMDN)	Wojtczak, J.A.; 2011	**Mean (SD):** In Diff = 51.33 (5.36) vs Easy = 57.55 (4.36); *P*-value = 0.052
	Hyomental distance in neutral positions HMDN (cm),	Andruszkiewicz, P.; 2016	**Mean (SD):** In Diff = 3.99 (0.56) vs Easy = 4.32 (0.42); P-value = 0.0014 **AUC** = 0.660, *P*-value = 0.002; **In cut-off = UK:** Sensitivity = 28.6%, Specificity = 94.4%, PPV = 37.5%, NPV = 91.8%
Length of the thyrohyoid membrane	Length of the thyrohyoid membrane	Wang, L.;2019	**Mean (SD):** In Diff = 1.83 (0.07) vs Easy = 2.07 (0.03); P-value< 0.001 **Odds ratio (OR)** = 0.22 (95% CI: 0.09–0.51)
	Thyrohyoid distance	Abraham, S.; 2018	**Mean (SD):** In Diff = 1.62 (0.44) vs Easy = 1.71 (0.62); *P*-value = 0.563
Tongue thickness	Tongue thickness (TT) (mm)	Xu, L.;2019	**Mean (SD):** In Diff = 61.4 (2.8) vs Easy = 54.6 (3.5); *P*-value< 0.001 **AUC** = 0.93 (95%CI: 0.88–0.98); **In cut-off > 58.65 mm**: Sensitivity = 0.85 (95% CI: 0.73–0.97), Specificity = 0.91 (95% CI: 0.85–0.98), PPV = 0.83 (95% CI: 0.71–0.98), NPV = 0.92 (95% CI: 0.86–0.98)
	Tongue thickness	Yadav, N.K.;2019	**Median (IQR):** In Diff = 6.1 (1.04) vs Easy = 5.30 (1.02); P-value = 0.001 **AUC** = 0.72 (95%CI: 0.62–0.81), **In cut-off = UK:** Sensitivity = 71.0%, Specificity = 72.0%
	Tongue thickness (cm)	Yao, W.; 2017 (a)	**Mean (SD):** In Diff = 6.4 (0.4) vs Easy = 5.9 (0.5); P-value< 0.001 **AUC** = 0.78 (95%CI: 0.77–0.80); **In cut-off > 6.1 cm**: Sensitivity = 0.75 (95% CI: 0.60–0.86), Specificity = 0.72 (95% CI: 0.70–0.74), PPV = 0.06 (95% CI: 0.04–0.08), NPV = 0.99 (95% CI: 0.99–1.0) **Odds ratio (OR) in cut-off** = 7.7 (95%CI: 3.9–16)
Condylar translation	Condylar translation (CT) (mm)	Xu, L.;2019	**Mean (SD):** In Diff = 10.5 (2.0) vs Easy = 12.8 (2.5); *P*-value< 0.001 **AUC** = 0.77 (95%CI: 0.67–0.86); **In cut-off < 11.05 mm**: Sensitivity = 0.70 (95% CI: 0.55–0.89), Specificity = 0.81 (95% CI: 0.72–0.90), PPV = 0.65 (95% CI: 0.50–0.80), NPV = 0.84 (95% CI: 0.76–0.93)
	Mandibular condylar mobility (mm)	Yao, W.; 2017 (b)	**Mean (SD):** In Diff = 9.2 (1.7) vs Easy = 13.7 (2.5); P-value< 0.001 **In Cut-off limited condylar translation:** Sensitivity = 0.81 (99% CI: 0.6–0.95), Specificity = 0.91 (99% CI: 0.87–0.94), PPV = 0.45 (99% CI: 0.29–0.62), NPV = 0.98 (99% CI: 0.96–1) **Odds ratio (OR) in limited condylar translation** = 40.4 (CI:13.5–121.4)
Anterior neck soft tissue at the of the Thyrohyoid membrane	Anterior neck soft tissue at the of the Thyrohyoid membrane	Adhikari, S.; 2011	**Mean (95% CI):** In Diff = 3.47 (2.88–4.07) vs Easy = 2.37 (2.29–2.44); *P*-value< 0.05
	Anterior neck thicknesses at the Thyrohyoid Membrane (THM)	Fulkerson, J.S.;2019	**Mean (SD):** In Diff = 2.0 (0.47) vs Easy = 2.14 (0.48); P-value = 0.304
	Distance from skin to the thyrohyoid membrane (STM) in neutral	Yadav, N.K.;2019	**Mean (SD):** In Diff = 1.58 (0.34) vs Easy = 1.93 (0.42); P-value< 0.001 **AUC** = 0.73 (95%CI: 0.63–0.83), **In cut-off = 2.03:** Sensitivity = 65.0%, Specificity = 69.0%
HMD distance between the hyoid bone and the posterior border of the symphisis menti	HMD distance between the hyoid bone and the posterior border of the symphisis menti: neutral	Petrișor, C.; 2018	**Mean (SD):** In Diff 4.04 (0.1) vs Easy 4.34 (0.32); P-value = 0.31
	HMD distance between the hyoid bone and the posterior border of the symphisis menti: ramped	Petrișor, C.; 2018	**Mean (SD):** In Diff 4.53 (0.1) vs Easy 5.17 (0.28); *P*-value = 0.03
	HMDR (the ratio between the HMD in the ramped position to that in neutral position)	Petrișor, C.; 2018	**Mean (SD):** In Diff 1.12 (0.001) vs Easy 1.2 (0.1); P-value = 0.02
Other indicators			
1	Ratios of tongue thickness to thyromental distance	Yao, W.; 2017 (a)	**Mean (SD):** In Diff = 0.94 (0.10) vs Easy = 0.80 (0.11); P-value< 0.001 **AUC** = 0.86 (95% CI: 0.84–0.87); **In cut-off > 0.87:** Sensitivity = 0.84 (95% CI: 0.71–0.93), Specificity = 0.79 (95% CI: 0.77–0.81), PPV = 0.09 (95% CI: 0.06–0.11), NPV = 1.0 (95% CI: 0.99–1.0) **Odds ratio (OR) in cut-off** = 20 (95% CI: 9.6–44.0)
2	Tongue cross-sectional area (TCSA) (cm2)	Andruszkiewicz, P.; 2016	**Mean (SD):** In Diff = 23.1 (3.57) vs Easy = 21.6 (3.09); *P*-value = 0.033 **AUC** = 0.622, *P*-value = 0.037; **In cut-off = UK:** Sensitivity = 9.1%, Specificity = 97.2%, PPV = 28.6%, NPV = 94.5%
3	Width of the tongue (cm)	Wang, L.;2019	**Mean (SD):** In Diff = 3.02 (0.05) vs Easy = 2.81 (0.03); P-value< 0.001
4	DSH + DSE	Martínez-García, A.;2020	**Mean (SD):** In Diff = 4.25 (0.45) vs Easy = 3.62 (0.77); *P*-value = 0.001 **AUC** = 0.75 (95%CI: 0.62–0.89), *P* = 0.001**; In cut-off = UK:** Sensitivity =81.25%، Specificity =70.59%، PPV =56.52%، NPV =88.89% **In cut-off = 4.5:** Sensitivity = 37.5% (95% CI: 10.7–64.4), Specificity = 82.4% (95% CI: 68.1–96.6), PPV = 50.0% (95% CI: 17.5–82.5), NPV = 73.7% (95% CI: 58.2–89.0)
5	DSE – DSG	Martínez-García, A.;2020	**Mean (SD):** In Diff = 1.83 (0.54) vs Easy = 1.24 (0.46); *P*-value = 0.001 **AUC** = 0.82 (95%CI: 0.68–0.96), *P* = 0.001**; In cut-off = UK:** Sensitivity =81.25%، Specificity =52.94%، PPV =44.83%، NPV =85.71% **In cut-off = 1.9:** Sensitivity = 68.8% (95% CI: 42.9–94.6), Specificity = 91.2% (95% CI: 80.2–100), PPV = 78.6% (95% CI: 53.5–100), NPV = 86.1% (95% CI: 73.4–98.8)
6	Thickness of the base of the tongue	Wang, L.;2019	**Mean (SD):** In Diff = 2.85 (0.09) vs Easy = 2.56 (0.04); *P*-value = 0.002 **Odds ratio (OR)** = 2.51 (95% CI: 1.38–4.55)
7	Angle between the epiglottis and glottis (°)	Wang, L.; 2019	**Mean (SD):** In Diff = 54.97 (4.93) vs Easy = 47.49 (4.17); *P*-value< 0.001 **Odds ratio (OR)** = 0.62 (95% CI: 0.54–0.71) **AUC** = 0.902 (95%CI: 0.846–0.957), **In cut-off = 50°:** Sensitivity = 81.0%, Specificity = 89.0
8	Distance from skin to hyoid bone (SHB) in sniffing	Yadav, N.K.; 2019	**Mean (SD):** In Diff = 0.53 (0.20) vs Easy = 0.73 (0.23); P-value< 0.001 **AUC** = 0.73 (95%CI: 0.63–0.84), **In cut-off = 0.77:** Sensitivity = 68.0%, Specificity = 72.0%
9	Distance from skin to the thyrohyoid membrane (STM) in sniffing	Yadav, N.K.; 2019	**Mean (SD):** In Diff = 1.54 (0.35) vs Easy = 1.84 (0.39); P-value< 0.001 **AUC** = 0.70 (95%CI: 0.60–0.81), **In cut-off = 1.9:** Sensitivity = 65.0%, Specificity = 63.0%
10	Tongue width (TW) (cm)	Andruszkiewicz, P.; 2016	**Mean (SD):** In Diff = 5.21 (0.45) vs Easy = 5.14 (0.46); P-value = 0.485 **AUC** = 0.589, P-value = 0.483; **In cut-off = UK:** Sensitivity = 9.1%, Specificity = 76.3%, PPV = 4.5%, NPV = 87.1%
11	Tongue thickness-to-oral cavity height ratio (TT/OCH)	Andruszkiewicz, P.; 2016	**Mean (SD):** In Diff = 0.84 (0.04) vs Easy = 0.83 (0.04); P-value = 0.347 **AUC** = 0.513, P-value = 0.339; **In cut-off = UK:** Sensitivity = 31.8%, Specificity = 68.4%, PPV = 11.1%, NPV = 89.0%
12	Floor of the mouth muscle Cross-sectional area (FFM CSA) (cm2)	Andruszkiewicz, P.; 2016	**Mean (SD):** In Diff = 4.75 (1.04) vs Easy = 4.48 (0.80); P-value = 0.1464 **AUC** = 0.571, P-value = 0.148 **In cut-off = UK:** Sensitivity = 9.1%, Specificity = 93.8%, PPV = 15.4%, NPV = 89.2%
13	Ratio of the Depth of the pre-epiglottic space (Pre-E) to the distance (cm)	Reddy, P.B.; 2016	**Mean (SD):** In CL1 = 0.98 (0.25), Range = 0.43–0.74/ CL2 = 1.08 (0.21), Range = 0.59–1.66/ CL3 = 1.04 (0.22), Range = 0.59–1.4; P-value = 0.134
14	Distance from the epiglottis to the mid-point of the distance between the vocal cords (E-VC) (cm)	Reddy, P.B.; 2016	**Mean (SD):** In CL1 = 0.96 (0.30), Range = 0.42–1.72/ CL2 = 0.89 (0.23), Range = 0.57–1.58/ CL3 = 0.84 (0.19), Range = 0.57–1.25; P-value = 0.214
15	Hyomental distance (HMD) to Hyoid bone (HB) ratio	Fulkerson, J.S.;2019	**Mean (SD):** In Diff = 5.05 (1.73) vs Easy = 6.12 (2.7); P-value = 0.139
16	Hyomental distance to Thyrohyoid membrane (THM) ratio	Fulkerson, J.S.;2019	**Mean (SD):** In Diff = 2.55 (1.03) vs Easy = 2.62 (0.85); P-value = 0.749
17	Hyomental distance to vocal cords (VC) ratio	Fulkerson, J.S.;2019	**Mean (SD):** In Diff = 6.87 (2.62) vs Easy = 8.25 (2.92); P-value = 0.080
18	Hyomental distance to Thyrohyoid membrane (THM) ratio	Fulkerson, J.S.;2019	**Mean (SD):** In Diff = 0.49 (0.14) vs Easy = 0.47 (0.17); P-value = 0.606
19	Hyoid bone to vocal cords ratio	Fulkerson, J.S.;2019	**Mean (SD):** In Diff = 1.37 (0.46) vs Easy = 1.47 (0.59); P-value = 0.482
20	Thyrohyoid membrane to vocal cords ratio	Fulkerson, J.S.;2019	**Mean (SD):** In Diff = 2.85 (0.82) vs Easy = 3.3 (1.24); P-value = 0.174
21	Thickness of the lateral pharyngeal	Wang, L.; 2019	**Mean (SD):** In Diff = 0.91 (0.04) vs Easy = 0.94 (0.02); P-value = 0.432 **Odds ratio (OR)** = 2.51 (95% CI: 1.38–4.55)
22	Thickness of submental region	Abraham, S.; 2018	**Mean (SD):** In Diff = 1.31 (0.27) vs Easy = 1.11 (0.32); P-value = 0.057
23	Epiglottis to hyoid bone distance	Abraham, S.; 2018	**Mean (SD):** In Diff = 1.87 (0.36) vs Easy = 1.8 (0.54); P-value = 0.695
24	Skin pad thickness to thyroid cartilage	Abraham, S.; 2018	**Mean (SD):** In Diff = 1.29 (0.48) vs Easy = 1.08 (0.49); P-value = 0.191
25	Ratio of the pre-epiglottis space distance and the distance between epiglottis and vocal folds (Pre-E/aVF)	Chan, S.M.M.; 2018	**AUC** = 0.648, P-value = 0.044; **In cut-off > 1:** Sensitivity = 79.5%, Specificity = 39.2%, PPV = 40.8%, NPV = 78.4%, PLR = 1.31, NLR = 0.52;
26	Pre-epiglottic area (PEA), cm2	Falcetta, S.; 2018	**AUC** = 0.93 (95% CI: 0.89–0.95); **In cut-off = 5.04:** Sensitivity = 85%, Specificity = 88%
27	Ability to view the hyoid bone in sublingual ultrasound	Hui, C.M.; 2014	**Number (%):** In Dif.: not seen = 8 (72.7), seen = 3 (27.3) vs Easy: not seen = 3 (3.4), seen = 86 (96.6); P-value< 0.001 Sensitivity = 70.0%, Specificity = 97.0%, PLR = 21.6, NLR = 0.28
28	Base of the tongue	Adhikari, S.; 2011	No-significant difference

*AUC* Area under the curve of ROC, *BMI* Body mass index, *CL* Cormack-Lehane grade, *CI* Confidence interval, *Diff* Difficult intubation group, *F* Female, *M* male, *NPV* Negative predictive value, *NLR* Negative likelihood ratio, *OR* Odds ratio, *PPV* Positive predictive value, *PLR* Positive likelihood, *SD* Standard deviation, *UK* Un-Known

#### Thickness of the anterior neck soft tissue at the vocal cords level

**Fig. 3:**
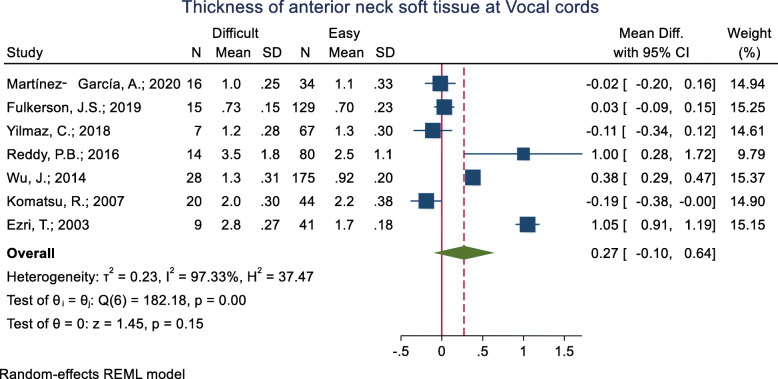
Forest-plot and pooled mean difference of thickness of anterior neck soft tissue at Vocal cords in difficult and easy intubation group

This criterion has been studied in nine studies (Table 3). The mean of this index was assessed in eight of these nine studies while the other one did not present the raw mean (±SD) data in the two groups and sufficed to report that the difference between the difficult and easy groups was not significant. As for the remaining eight studies, the mean thickness of the anterior neck soft tissue at the vocal cords was significantly higher in the difficult than the easy group in three studies. In three other studies, the mean of the index was higher in the easy group, and in one study, it was not statistically significant. The pooled mean difference of the anterior neck soft tissue at the vocal cords based on the meta-analysis results was 0.27 cm higher in the difficult than the easy group and this difference was marginally significant (*p* = 0.150) (Fig. 3). The AUC was reported as 0.47, 0.54 and 0.85 in three studies. In one study with an unknown cut-off point, the sensitivity and specificity were reported as 53 and 66%, respectively.

#### Anterior neck soft tissue at the hyoid bone level

**Fig. 4:**
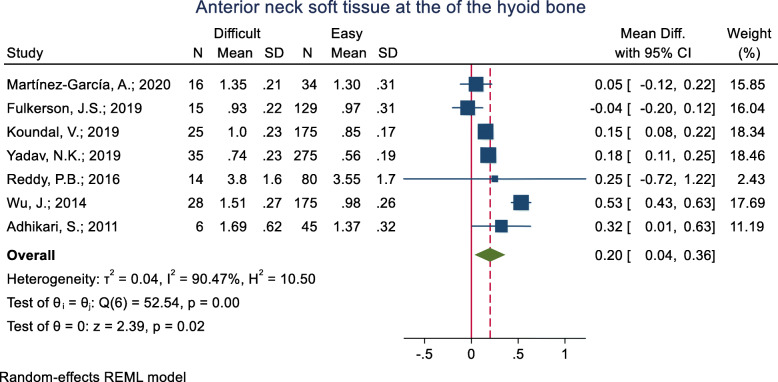
Forest-plot and pooled mean difference of anterior neck soft tissue at the of the hyoid bone in difficult and easy intubation group

This index was assessed in eight studies (Table 3). Seven studies assessed the mean difference and five of them showed that the mean of the anterior neck soft tissue at the hyoid bone was significantly higher in the difficult intubation group compared to the easy group. The pooled mean difference of this index based on the meta-analysis was 0.20 cm higher in the difficult than the easy group and this difference was significant (*p* < 0.001) (Fig. 4). The AUC of the anterior neck soft tissue at the hyoid bone was reported as 0.559 to 0.92 in five studies. Two studies reported the sensitivity and specificity of this index but their cut-off point was unknown. In two studies, the optimal cut-off point was calculated as 0.66 (sensitivity = 68.0 and specificity = 69.0) and 0.99 (sensitivity = 48.0 and specificity = 82.0).

#### Hyomental distance (HMD) with the neck extended

**Fig. 5:**
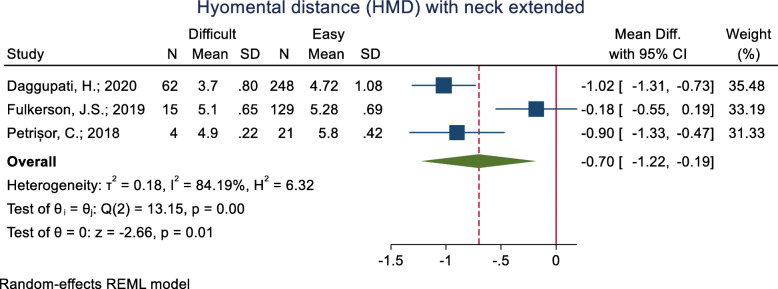
Forest-plot and pooled mean difference of hyomental distance (HMD) with neck extended in difficult and easy intubation group

This index was assessed in three studies (Table 3). All of the three studies assessed the mean difference and none of them not showed a significant mean difference between the difficult and easy intubation groups. The pooled mean difference of this index based on the meta-analysis was 0.70 cm higher in the difficult than the easy group and this difference was significant (*p* < 0.001) (Fig. 5).

#### Hyomental distance ratio (HMDR)

**Fig. 6:**
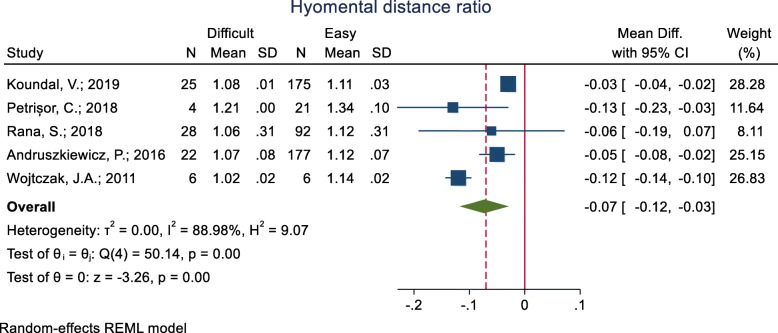
Forest-plot and pooled mean difference of hyomental distance ratio (HMDR) in difficult and easy intubation group

This index was assessed in five studies (Table 3), and in all of them, the mean HMDR was significantly lower in the difficult group compared to the easy group. The pooled mean difference of HMDR based on the meta-analysis was 0.07 cm lower in the difficult than the easy group and this difference was significant (*p* < 0.001) (Fig. 6). The AUC of this index was reported as 0.71, 0.76 and 0.87 in three studies. In two studies, the optimal cut-off point was calculated as 1.085 (sensitivity = 75.0 and specificity = 85.3) and 1.087 (sensitivity = 65.0 and specificity = 77.0).

#### Ratio of the pre-epiglottic space (pre-E) and epiglottis vocal cord (E-VC) distances

**Fig. 7:**
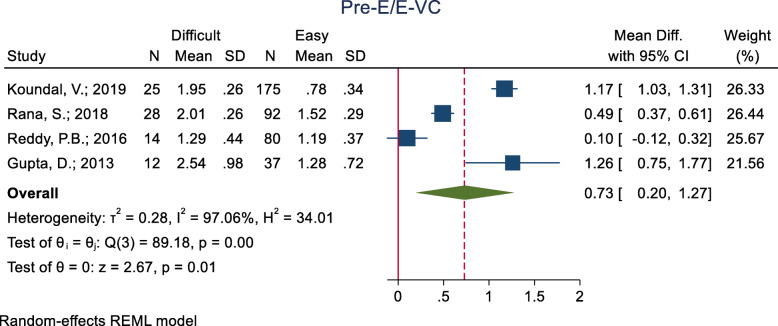
Forest-plot and pooled mean difference of ratio of Pre-E and E-VC distances in difficult and easy intubation group

This index was assessed in four studies (Table 3) and all of them showed a significantly higher mean Pre-E/E-VC in the difficult than the easy group. The pooled mean difference of the ratio of Pre-E and E-VC distances based on the meta-analysis was 0.73 cm higher in the difficult than the easy group and this difference was significant (p < 0.001) (Fig. 7). The AUC of this index was reported as 0.868 and 0.871 in two studies. In two studies, the optimal cut-off point was 1.77 (sensitivity = 82.0 and specificity = 80.0) and 1.77 (sensitivity = 82.0 and specificity = 80.0).

#### Anterior neck soft tissue at the thyroid isthmus

**Fig. 8:**
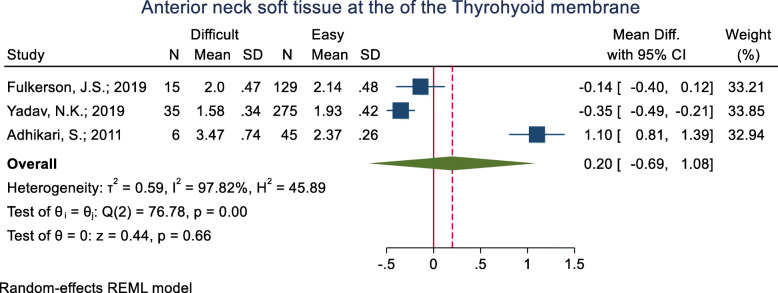
Forest-plot and pooled mean difference of anterior neck soft tissue at thyroid isthmus in difficult and easy intubation group

This index was examined in three studies (Table 3). One of these studies, however, did not present the raw mean (±SD) data in the two groups and only reported that the groups were not significantly different in this regard. Also, the two remaining studies did not show a significant mean difference between the two groups in anterior neck soft tissue at the thyroid isthmus. The pooled mean difference of this index based on the meta-analysis was not significantly different (*p* = 0.880) (Fig. 8).

#### Anterior neck soft tissue at the suprasternal notch

**Fig. 9:**
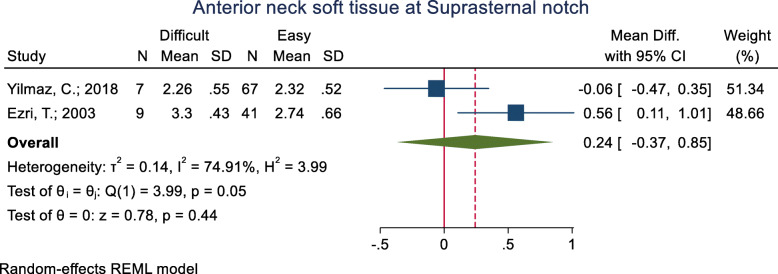
Forest-plot and pooled mean difference of anterior neck soft tissue at Suprasternal notch in difficult and easy intubation group

This index was assessed in three studies (Table 3). One of these studies did not present the raw mean (±SD) data in the two groups and only reported that the groups were not significantly different in this regard. As for the two remaining studies, the mean of this index was significantly higher in the difficult intubation group than the easy group in one study [33.0 (4.3) vs. 27.4 (6.6) mm; *p* = 0.013], while the other study did not show any significant differences between the groups in this regard (*p* = 0.931). The pooled mean difference of this index based on the meta-analysis was 0.24 cm higher in the difficult than the easy group, although this difference was not significant (*p* = 0.440) (Fig. 9).

#### Tongue volume

**Fig. 10:**
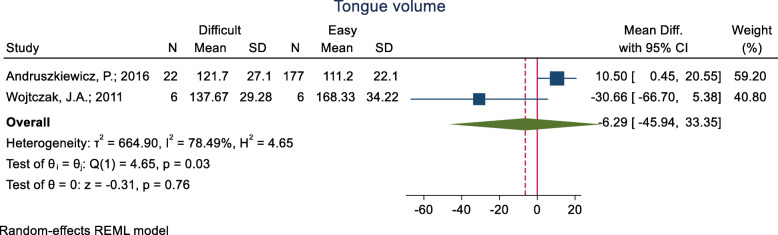
Forest-plot and pooled mean difference of tongue volume in difficult and easy intubation group

This index has been assessed in three studies (Table 3), in which, the mean difference of tongue volume reported in two group and have inconsistent result. The pooled mean difference of this index based on the meta-analysis of the two studies was 6.29 cm^3^ lower in the difficult than the easy group, although this difference was not significant (*p* = 0.760) (Fig. 10). The AUC of tongue volume was reported as 0.626 in one study, and in the other study with the cut-off point of 100 cm^3^, the sensitivity and specificity were reported as 66.7 and 62.7%, respectively.

#### Floor of the mouth muscle volumes

**Fig. 11:**
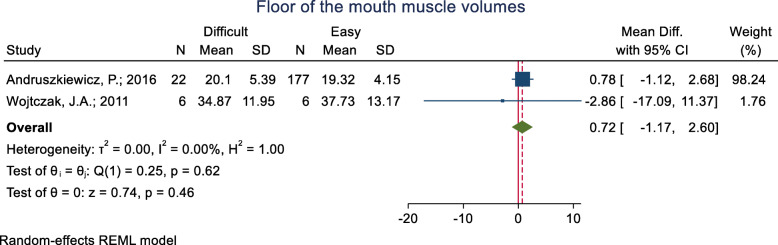
Forest-plot and pooled mean difference of floor of the mouth muscle volumes in difficult and easy intubation group

This index was assessed in three studies (Table 3). The mean difference in the floor of the mouth muscle volumes was reported in two groups and it was not significant. The pooled mean difference of this index based on the meta-analysis of two studies was also not significant (*p* = 0.460) (Fig. 11). The AUC of the floor of the mouth muscle volumes was reported as 0.559 in one study. Two studies reported a sensitivity and specificity for this index but their cut-off point was unknown.

#### Hyomental distance in the head positions (HMDE)

**Fig. 12:**
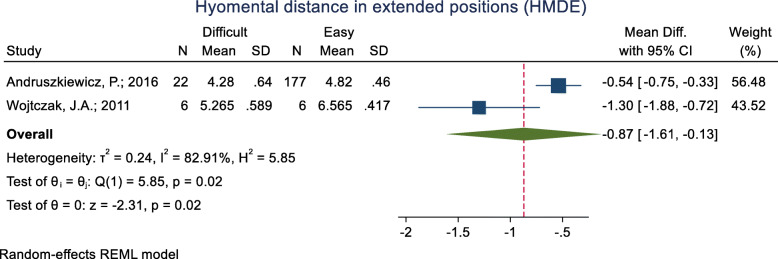
Forest-plot and pooled mean difference of hyomental distance in the head positions (HMDE) in difficult and easy intubation group

This index was assessed in two studies (Table 3), and in both of them, the mean HMDE was significantly lower in the difficult intubation group than the easy group. The pooled mean difference of this index based on the meta-analysis was 0.87 cm lower in the difficult than the easy group and the difference was significant (*p* = 0.020) (Fig. 12). The AUC of HMDE was reported as 0.758. The sensitivity and specificity of this index for an unknown cut-off point were 38.1 and 97.7% (Table 2).

#### Hyomental distance in the neutral positions (HMDN)

**Fig. 13:**
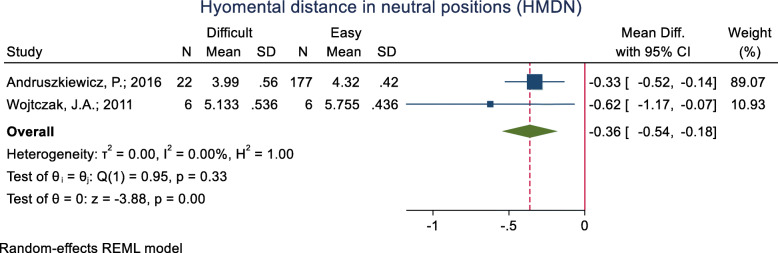
Forest-plot and pooled mean difference of hyomental distance in the neutral positions (HMDN) in difficult and easy intubation group

This index was assessed in two studies (Table 3), and in both of them, the mean HMDN was significantly lower in the difficult intubation group than the easy group. The pooled mean difference of this index based on the meta-analysis was 0.36 cm lower in the difficult than the easy group and this difference was significant (*p* < 0.001) (Fig. 13). The AUC of HMDE was reported as 0.66. The sensitivity and specificity of this index for an unknown cut-off point were 28.6 and 94.4% (Table 2).

#### Length of the thyrohyoid membrane

**Fig. 14:**
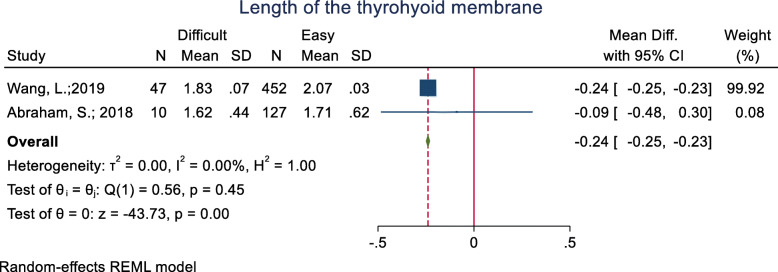
Forest-plot and pooled mean difference of length of the thyrohyoid membrane in difficult and easy intubation group

This index was assessed in two studies (Table 3), and in both of them, the mean length of the thyrohyoid membrane was lower in the difficult intubation group than the easy group. Meanwhile, the mean difference was significant in only one of the studies [Mean (SD): 1.83 (0.07 vs. 2.07 (0.03); *p* < 0.001]. The pooled mean difference of this index based on the meta-analysis was 0.24 cm lower in the difficult than the easy group and this difference was significant (*p* < 0.001) (Fig. 14).

#### Tongue thickness

**Fig. 15:**
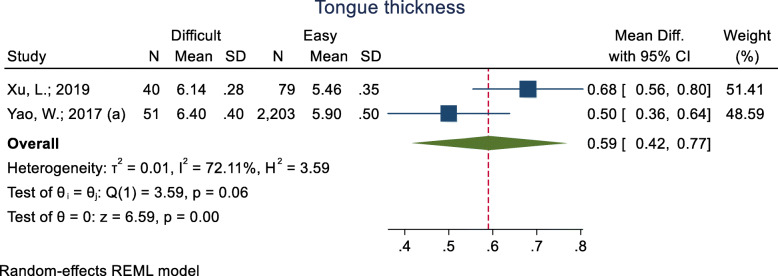
Forest-plot and pooled mean difference of tongue thickness in difficult and easy intubation group

This index was assessed in three studies (Table 3), and in all three, the mean (two study) and median (one study) of tongue thickness was higher in the difficult intubation group than the easy group. The pooled mean difference of this index based on the meta-analysis was 0.59 cm higher in the difficult than the easy group and this difference was significant (*p* < 0.001) (Fig. 15). The AUC of this index was reported as 0.72, 0.78 and 0.93. In two studies, the optimal cut-off point was calculated as 5.87 (sensitivity = 85.0 and specificity = 91.0) and 6.1 (sensitivity = 75.0 and specificity = 72.0).

#### Condylar translation

**Fig. 16:**
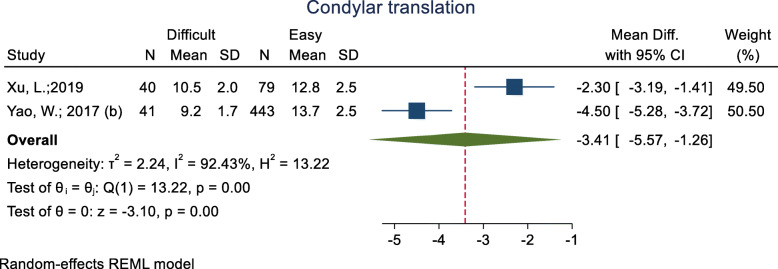
Forest-plot and pooled mean difference of condylar translation in difficult and easy intubation group

This index was assessed in two studies (Table 3) and all of them showed that the mean condylar translation was significantly lower in the difficult intubation group than the easy group. The pooled mean difference of this index based on the meta-analysis was 3.41 cm lower in the difficult than the easy group and this difference was significant (p < 0.001) (Fig. 16). The AUC of this index was reported as 0.77 in one study, and its sensitivity and specificity with a 11.05-mm cut-off point were 0.70 and 0.81. In another study, the sensitivity and specificity were 0.81 and 0.91, respectively.

#### Anterior neck soft tissue at the thyrohyoid membrane

**Fig. 17:**
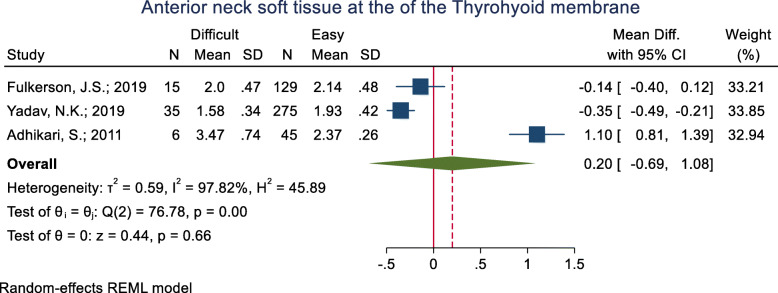
Forest-plot and pooled mean difference of anterior neck soft tissue at the of the thyrohyoid membrane in difficult and easy intubation group

This index was assessed in three studies (Table 3). One of them revealed the mean anterior neck soft tissue at the thyrohyoid membrane to be significantly higher in the difficult intubation group than the easy group and one study showed the opposite; the other study showed non-significant differences between the two groups. The pooled mean difference of this index based on the meta-analysis was 0.20 cm lower in the difficult than the easy group and this difference was significant (p < 0.001) (Fig. 17). The AUC of this index was reported as 0.73 in one study. Its sensitivity and specificity with a 2.03 cut-off point were 65.0 and 69.0% (Table 2).

#### HMD distance between the hyoid bone and the posterior border of the symphysis menti

This index was assessed in one study (Table 3) in the form of three US indicators: Neutral, ramped, and the ratio between HMD in the ramped position to HMD in the neutral position (HMDR). Of the three main indicators, the ramped criterion showed a significant difference between the difficult and easy intubation groups, such that the mean value of this criterion was significantly lower in the difficult intubation group than the group with easy intubation [4.53 (0.1) vs. 5.17 (0.28); *p* = 0.03]. Also, the ratio between the HMD in the ramped position to that in the neutral position was significantly lower in the difficult intubation group (*p* = 0.02) (Table 2).

#### Other indicators

A total of 28 US indicators were examined in 11 studies (Table 3). However, only one study was found for each of the following 28 indicators.

1. Ratios of tongue thickness to thyromental distance
2. Tongue cross-sectional area (TCSA) (cm2)
3. Width of the tongue (cm)
4. DSH + DSE
5. DSE – DSG
6. Thickness of the base of the tongue
7. Angle between the epiglottis and glottis (°)
8. Distance from skin to hyoid bone (SHB) in sniffing
9. Distance from skin to the thyrohyoid membrane (STM) in sniffing
10. Tongue width (TW) (cm)
11. Tongue thickness-to-oral cavity height ratio (TT/OCH)
12. Floor of the mouth muscle Cross-sectional area (FFM CSA) (cm2)
13. Ratio of the depth of the pre-epiglottic space (Pre-E) to the distance (cm)
14. Distance from the epiglottis to the mid-point of the distance between the vocal cords (E-VC) (cm)
15. Hyomental distance (HMD) to Hyoid bone (HB) ratio
16. Hyomental distance to Thyrohyoid membrane (THM) ratio
17. Hyomental distance to vocal cords (VC) ratio
18. Hyomental distance to thyrohyoid membrane (THM) ratio
19. Hyoid bone to vocal cords ratio
20. Thyrohyoid membrane to vocal cords ratio
21. Thickness of the lateral pharyngeal
22. Thickness of submental region
23. Epiglottis to hyoid bone distance
24. Skin pad thickness to thyroid cartilage
25. Ratio of the pre-epiglottis space distance and the distance between epiglottis and vocal folds (Pre-E/aVF)
26. Pre-epiglottic area (PEA), cm2
27. Ability to view the hyoid bone in sublingual ultrasound
28. Base of the tongue

Out of a total of 28 US indicators, the mean difference for 24 was assessed. The mean of seven indicators (numbers 1 to 7 of the “other indicators” list) was significantly higher in the difficult intubation group than the easy group (*p* < 0.05). The mean of two indicators (8: Distance from the skin to the hyoid bone (SHB) in sniffing, and 9: Distance from the skin to the thyrohyoid membrane (STM) in sniffing) was significantly lower in the difficult intubation group than the easy group, and the mean difference was not significant between the difficult and easy intubation groups for 15 indicators (numbers 10 to 24 of the “other indicators” list).

The AUC was reported for 12 indicators. The AUC was between 0.622 and 0.589 and non-significant for four indicators (2, 10, 11, and 12 of the “other indicators” list) and was between 0.648 and 0.930 and statistically significant (p < 0.05) for nine indicators (1, 4, 5, 7, 8, 9, 25, and 26 of the list). Sensitivity and specificity were reported for 12 indicators (1, 2, 4, 5, 7 to 12, 25, and 26 of the list), although the cut-off point was unknown for four indicators (2, and 10 to 12 of the list).

The ability to view the hyoid bone in the sublingual US index is a special issue to discuss; as its seen frequency in both difficult and easy group were ambiguous. This index had a significantly different distribution; however, its seen in difficult group was lower than easy intubation group (27.3% vs. 96.6%, *p* < 0.001). Also, the sensitivity and specificity of this index were 70 and 97%, respectively (Table 2).

No raw data was presented in any of the two groups for the base of the tongue index, and only a non-significant difference was reported between the difficult and easy groups in this index (Table 2).

## Discussion

This systematic review showed that US can be used for predicting difficult airway. The skin thickness at the epiglottis and hyoid levels, HMD, and HMDR were found to be correlated with difficult laryngoscopy in the meta-analysis. Many other indicators, including many ratios, are also proposed to accurately predict difficult intubation, although there are no external validation studies on them.

To have a clear visualization during direct laryngoscopy, many factors, such as mouth opening, oropharyngeal anatomy, mandibular space, neck motility, and performer’s skill are involved [32]. Many bedside assessment methods are used to evaluate the aforementioned aspects. For example, the Mallampati score evaluates the tongue size relative to the oropharyngeal space [33]. While these methods are widely used, their performances are under question. Using other tools, such as imaging studies (computed tomography (CT) scan and X-ray), is also limited due to radiation hazards, costs, and logistics [34].

US is used in various aspects of airway management and for many purposes, such as the prediction of pediatric ETT size, confirmation of correct placement of ETT, guidance of percutaneous tracheostomy and cricothyroidotomy, and confirmation of proper laryngeal mask airway position [35, 36]. In addition, US has been proposed to also measure some indicators that reflect the intubation-related anatomy directly or indirectly. This widely-available instrument can be easily applied and learned by clinicians. A meta-analysis study showed a similar performance between imaging studies and US in predicting difficult airways. It reported the overall accuracy of US as 0.89. The study did not assess different indicators separately and included studies including heterogeneity caused by differences in design and implementation [34].

It has been hypothesized that increased anterior neck soft tissue thickness can impair the mobility of the pharyngeal structures during laryngoscopy [30]. This distance can be measured via US machines in various levels, including the vocal cords, thyroid isthmus, and suprasternal notch, and hyoid bone. The results are conflicting among studies in this regard. While the pooled results were not significant at the vocal cords, thyroid isthmus, and suprasternal notch levels, it can be relied on at the hyoid bone and epiglottis levels. The difference is 0.2 cm at hyoid bone level and more than 0.6 mm with different cut-offs in epigglotic region. In a study by Yadav et al., many of the aforementioned distances were assessed in different positions, such as the sniffing position. The authors stated that the results were significant in many of the sites, though with low accuracy [13]. From another perspective, this soft tissue thickness could also be a presentation of a high BMI that is complicating orotracheal intubation.

Hyomental distance is considered an important factor for displacing the tongue during laryngoscopy [23]. Intubation might be more difficult in shorter distances that might present large mandibular size and its proximity to hyoid bone. This distance can be assessed in different positions. Petrișor et al. proposed that this distance is most accurate in hyperextension position among the obese in comparison with the neutral or ramped positions. While the study proposed a sensitivity of 100% for all the positions, specificity was 71.4% in hyperextension [37]. Our study showed that the difference in distance is less than 4 mm in the neutral position and is increased to 8 mm in the extension position. Therefore, its applicability should be further tested before clinical recommendations can be made. HMDR was first described among rheumatoid arthritis patients [38]. The distance between the hyoid bone and occipital bone remains constant during extension/flexion of the neck due to stylohyoid ligament. By neck extension just below the occiput, the mentum moves away from the hyoid bone, which increases the hyomental distance. It has been proposed that without this increase, lower cervical spine extension alone would take the larynx and glottis out of line of view by displacing the laryngeal structure forward [29]. According to the results, the ratio has a good specificity and fair accuracy.

Gupta et al. developed an oblique view for airway sonography [28]. By tilting the probe midline in the submandibular area caudally, they obtained a view that bisected the epiglottis and posterior-most part of the vocal folds. Using this view, they calculated the Pre-E and E-VC ratio. With an acceptable accuracy, both of the studies proposed 1.77 as the optimal cut-off and the meta-analysis showed significant differences between the difficult and easy intubation groups.

During the introduction of laryngoscope into the oral cavity, since the blade is positioned on the tongue, the tongue anatomy is important for better glottis view [33]. In addition to tongue thickness, it has been suggested to also calculate the tongue volume using a cross-sectional area of the tongue (at the midsagittal), multiplied by its width [29]. This method overestimates the tongue volume due to cautious tongue measurement along all its lengths from the mentum to the hyoid bone. Two studies on tongue volume yielded conflicting results; in the general public, tongue volume and area were a predictor of difficult intubation [23]. Meanwhile, in a study with a small sample of obese patients, there was no significant difference in this regard [29]. Pooling the data showed that while the difference between the two groups was 6 cm^3^, the accuracy was not acceptable. Concerning tongue thickness, while there is a fair accuracy, the measurements in the difficult intubation group failed to demonstrate differences in comparison with the easy ones. This study also showed that measuring the floor of the mouth muscle volume is inaccurate for difficult intubation prediction. Further studies are required on the measurement of the tongue width and thickness and their ratios to other parts.

### Limitations

There are several reasons for the conflicting results and the heterogeneity among the reviewed studies. On the one hand, there are patients with different baseline characteristics (e.g., BMI and ethnic background). On the other hand, while the gold standard among studies is usually the Cormack and Lehane score, this objective index is assessed in different conditions (e.g., with or without the Backward, Upward, Rightward Pressure (BURP) maneuver) [30] and by different assessors. In addition, US is operator-dependent and there are some variabilities due to the level of operator expertise and machine properties.

## Conclusion

To conclude, this systematic review and meta-analysis showed that US can be used to predict difficult airways. Nonetheless, its application should be carefully assessed in other settings before making any recommendations.

## Data Availability

The dataset is available following any request send to the correspondence.

## Appendix

**Table 4:** Search Strategy. Search strategy used in PubMed database – Search date: April 28, 2020

Search	Query
#1	(Ultrason* [Title/Abstract] OR Ultrasonic [Title/Abstract] OR Ultrasoun* [Title/Abstract] OR ultrasond [Title/Abstract] OR ultrasonography [Title/Abstract] OR Ultraso* measurement [Title/Abstract] OR Ultraso* investigation [Title/Abstract] OR Ultraso* parameter [Title/Abstract] OR Sono* measurement [Title/Abstract] OR sono* investigation [Title/Abstract] OR sono* parameter [Title/Abstract] OR Sonograph [Title/Abstract] OR sonography [Title/Abstract] OR ultrasonography [Title/Abstract] OR ultrasonographic [Title/Abstract]) OR (Cormack-lehane [Title/Abstract] OR “Cormack lehane” [Title/Abstract] OR hyposmia [Title/Abstract] OR hypoxia [Title/Abstract])
#2	airway evaluat* [Title/Abstract] OR airway manag* [Title/Abstract] OR airway invest* [Title/Abstract] OR Difficult Laryngoscopy [Title/Abstract] OR laryngoscop* [Title/Abstract] OR difficult airway [Title/Abstract] OR difficult intubation [Title/Abstract] OR intub* [Title/Abstract] OR endotracheal tube [Title/Abstract] OR endotracheal intubation [Title/Abstract] OR tracheal intubation [Title/Abstract] OR orotracheal intubation [Title/Abstract]
#3	#1 AND #2

## Abbreviations

AUC: Area under the curve
BMI: Body mass index
BURP: Backward, Upward, Rightward Pressure
ETT: Endotracheal tube
E-VC: Epiglottis-vocal cords
ED: Emergency department
HMD: Hyomental distance
HMDE: Hyomental distance in the head positions
HMDN: Hyomental distance in the neutral positions
HMDR: Hyomental distance ratio
OR: Odds ratio
PRISMA: Preferred Reporting Items for Systematic Reviews and Meta-Analyses
RSI: Rapid sequence intubation
SD: Standard deviation
US: Ultrasonography

## References

[1] Khan ZH, Kashfi A, Ebrahimkhani E (2003). A comparison of the upper lip bite test (a simple new technique) with modified Mallampati classification in predicting difficulty in endotracheal intubation: a prospective blinded study. Anesth Analg.

[2] Al Ramadhani S, Mohamed L, Rocke D, Gouws E, Ramadhani S (1996). Sternomental distance as the sole predictor of difficult laryngoscopy in obstetric anaesthesia. Br J Anaesth.

[3] Shiga T (2005). Wajima Zi, Inoue T, Sakamoto a. predicting difficult intubation in apparently Normal PatientsA meta-analysis of bedside screening test performance. Anesthesiology.

[4] Karkouti K, Rose DK, Ferris LE, Wigglesworth DF, Meisami-Fard T, Lee H (1996). Inter-observer reliability of ten tests used for predicting difficult tracheal intubation. Can J Anaesth.

[5] Adhikari S, Zeger W, Schmier C, Crum T, Craven A, Frrokaj I, Pang H, Shostrom V (2011). Pilot study to determine the utility of point-of-care ultrasound in the assessment of difficult laryngoscopy. Acad Emerg Med.

[6] Lambert AS, Tousignant CP (2013). Anesthesia and ultrasound: riding the waves. Can J Anesthesia/Journal canadien d'anesthésie.

[7] Daggupati H, Maurya I, Singh RD, Ravishankar M (2020). Development of a scoring system for predicting difficult intubation using ultrasonography. Ind J Anaesthesia.

[8] Martínez-García A, Guerrero-Orriach JL, Pino-Gálvez MA. Ultrasonography for predicting a difficult laryngoscopy. Getting closer. Journal of clinical monitoring and computing. 2020. 10.1007/s10877-020-00467-1 31993893

[9] Fulkerson JS, Moore HM, Lowe RF, Anderson TS, Lucas LL, Reed JW (2019). Airway sonography fails to detect difficult laryngoscopy in an adult veteran surgical population. Trends in Anaesthesia Crit Care.

[10] Xu L, Dai S, Sun L, Shen J, Lv C, Chen X (2020). Evaluation of 2 ultrasonic indicators as predictors of difficult laryngoscopy in pregnant women: A prospective, double blinded study. Medicine.

[11] Koundal V, Rana S, Thakur R, Chauhan V, Ekke S, Kumar M (2019). The usefulness of point of care ultrasound (POCUS) in preanaesthetic airway assessment. Indian J Anaesthesia.

[12] Wang L, Feng YK, Hong L, Xie WL, Chen SQ, Yin P, Wu QP (2019). Ultrasound for diagnosing new difficult laryngoscopy indicator: a prospective, self-controlled, assessor blinded, observational study. Chin Med J.

[13] Yadav NK, Rudingwa P, Mishra SK, Pannerselvam S (2019). Ultrasound measurement of anterior neck soft tissue and tongue thickness to predict difficult laryngoscopy - an observational analytical study. Indian journal of anaesthesia..

[14] Abraham S, Himarani J, Mary Nancy S, Shanmugasundaram S, Krishnakumar Raja VB (2018). Ultrasound as an assessment method in predicting difficult intubation: a prospective clinical study. J Maxillofacial Oral Surg.

[15] Chan SMM, Wong WY, Lam SKT, Law WSS, Shiu WYY, Wong OF (2018). Use of ultrasound to predict difficult intubation in Chinese population by assessing the ratio of the pre-epiglottis space distance and the distance between epiglottis and vocal folds. Hong Kong J Emerg Med.

[16] Falcetta S, Cavallo S, Gabbanelli V, Pelaia P, Sorbello M, Zdravkovic I, Donati A (2018). Evaluation of two neck ultrasound measurements as predictors of difficult direct laryngoscopy: a prospective observational study. Eur J Anaesthesiol.

[17] Petrisor C, Szabo R, Constantinescu C, Prie A, Hagau N (2018). Ultrasound-based assessment of hyomental distances in neutral, ramped, and maximum hyperextended positions, and derived ratios, for the prediction of difficult airway in the obese population: a pilot diagnostic accuracy study. Anaesthesiology Intensive Ther.

[18] Rana S, Verma V, Bhandari S, Sharma S, Koundal V, Chaudhary SK (2018). Point-of-care ultrasound in the airway assessment: a correlation of ultrasonography-guided parameters to the Cormack-Lehane classification. Saudi J Anaesth.

[19] Yilmaz C, Karasu D, Dilektasli E, Taha A, Ozgunay SE, Korfali G (2018). An evaluation of ultrasound measurements of anterior neck soft tissue and other predictors of difficult laryngoscopy in morbidly obese patients. Bariatric Surgical Practice Patient Care.

[20] Parameswari A, Govind M, Vakamudi M (2017). Correlation between preoperative ultrasonographic airway assessment and laryngoscopic view in adult patients: a prospective study. J Anaesthesiol Clin Pharmacol.

[21] Yao W, Wang B (2017). Can tongue thickness measured by ultrasonography predict difficult tracheal intubation?. Br J Anaesth.

[22] Yao W, Zhou Y, Wang B, Yu T, Shen Z, Wu H, Jin X, Li Y (2017). Can mandibular condylar mobility Sonography measurements predict difficult laryngoscopy?. Anesth Analg.

[23] Andruszkiewicz P, Wojtczak J, Sobczyk D, Stach O, Kowalik I (2016). Effectiveness and validity of Sonographic upper airway evaluation to predict difficult laryngoscopy. J Ultrasound Med.

[24] Pinto J, Cordeiro L, Pereira C, Gama R, Fernandes HL, Assunção J (2016). Predicting difficult laryngoscopy using ultrasound measurement of distance from skin to epiglottis. J Crit Care.

[25] Reddy PB, Punetha P, Chalam KS (2016). Ultrasonography - a viable tool for airway assessment. Indian J Anaesthesia.

[26] Hui CM, Tsui BC (2014). Sublingual ultrasound as an assessment method for predicting difficult intubation: a pilot study. Anaesthesia..

[27] Wu J, Dong J, Ding Y, Zheng J (2014). Role of anterior neck soft tissue quantifications by ultrasound in predicting difficult laryngoscopy. Medical Science Monitor.

[28] Gupta D, Srirajakalidindi A, Ittiara B, Apple L, Toshniwal G, Haber H (2012). Ultrasonographic modification of Cormack Lehane classification for pre-anesthetic airway assessment. Middle East journal of anaesthesiology.

[29] Wojtczak JA (2012). Submandibular sonography: assessment of hyomental distances and ratio, tongue size, and floor of the mouth musculature using portable sonography. J Ultrasound Med.

[30] Komatsu R, Sengupta P, Wadhwa A, Akça O, Sessler DI, Ezri T, Lenhardt R (2007). Ultrasound quantification of anterior soft tissue thickness fails to predict difficult laryngoscopy in obese patients. Anaesth Intensive Care.

[31] Ezri T, Gewürtz G, Sessler DI, Medalion B, Szmuk P, Hagberg C, Susmallian S (2003). Prediction of difficult laryngoscopy in obese patients by ultrasound quantification of anterior neck soft tissue. Anaesthesia..

[32] Langeron O, Cuvillon P, Ibanez-Esteve C, Lenfant F, Riou B, Le Manach Y (2012). Prediction of difficult tracheal IntubationTime for a paradigm change. Anesthesiology.

[33] Mallampati SR, Gatt SP, Gugino LD, Desai SP, Waraksa B, Freiberger D, Liu PL (1985). A clinical sign to predict difficult tracheal intubation; a prospective study. Canadian Anaesthetists’ Society J.

[34] Ji C, Ni Q, Chen W (2018). Diagnostic accuracy of radiology (CT, X-ray, US) for predicting difficult intubation in adults: a meta-analysis. J Clin Anesth.

[35] Kajekar P, Mendonca C, Gaur V (2010). Role of ultrasound in airway assessment and management. International Journal of Ultrasound & Applied Technologies in Perioperative Care.

[36] Gupta PK, Gupta K, Dwivedi AND, Jain M. Potential role of ultrasound in anesthesia and intensive care. Anesthesia, essays and researches. 2011;5(1):11. 10.4103/0259-1162.84172 PMC4173359 25885294

[37] Petrișor C, Szabo R, Constantinescu C, Prie A, Hagău N (2018). Ultrasound-based assessment of hyomental distances in neutral, ramped, and maximum hyperextended positions, and derived ratios, for the prediction of difficult airway in the obese population: a pilot diagnostic accuracy study. Anaesthesiology Intensive Ther.

[38] Takenaka I, Iwagaki T, Aoyama K, Ishimura H, Kadoya T (2006). Preoperative evaluation of extension capacity of the Occipitoatlantoaxial complex in patients with rheumatoid ArthritisComparison between the Bellhouse test and a new method, Hyomental distance ratio. Anesthesiology.

